# Co-delivery of paclitaxel and gemcitabine *via* a self-assembling nanoparticle for targeted treatment of breast cancer†

**DOI:** 10.1039/c9ra00276f

**Published:** 2019-02-13

**Authors:** Meng Lei, Sijia Sha, Xueyuan Wang, Jia Wang, Xiao Du, Hang Miao, Hui Zhou, Enhe Bai, Jingmiao Shi, Yongqiang Zhu

**Affiliations:** College of Science, Nanjing Forestry University No. 159 Longpan Road Nanjing 210037 PR China; College of Life Science, Nanjing Normal University No. 1 Wenyuan Road Nanjing 210046 PR China zhyqscu@hotmail.com; Department of Pharmaceutics, School of Pharmacy, China Pharmaceutical University Nanjing 210009 PR China duxiaojianai@126.com; Jiangsu Chia Tai Fenghai Pharmaceutical Co. Ltd. No. 9 Weidi Road Nanjing 210046 PR China

## Abstract

Multi-functional nanoparticles can be used to improve the treatment index and reduce side effects of anti-tumor drugs. Herein, we developed a kind of multi-functional and highly biocompatible nanoparticle (NP) loaded with folic acid (FA), paclitaxel (PTX) and gemcitabine (GEM) *via* self-assembly to target cancer cells. The transmission electron microscopy (TEM) results showed that multi-functional FA targeting nanoparticles (MF-FA NPs) exhibited spherical morphology and favorable structural stability in aqueous solution. In addition, NPs (MF-FA NPs and MF NPs) exhibited comparable proliferation inhibition to breast cancer cell 4T1 compared with the pure drug. In *in vivo* antitumor studies, NPs showed an obviously enhanced anti-tumor efficacy compared with the pure drug. Furthermore, MF-FA NPs displayed higher tumor growth inhibition than MF NPs due to the specific targeting of FA to cancer cells. Consequently, the novel MF-FA NPs could be used as a potential chemotherapeutic formulation for breast cancer therapy.

## Abstract 2

Preparation of MF-FA nanoparticles and the release behavior of drugs in tumor cells.

## Graphical abstract

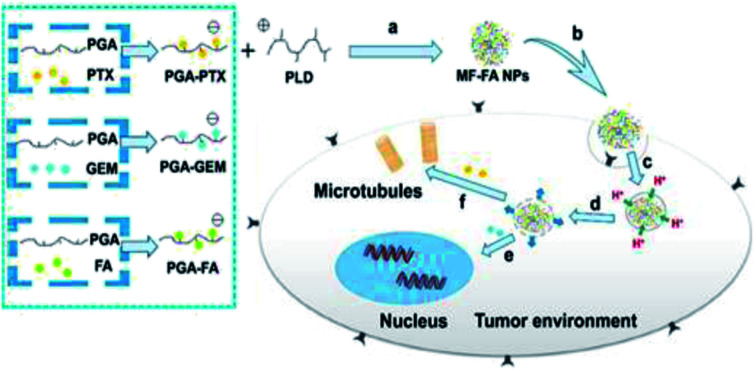

## 1. Introduction

As one of the most common types of cancer, breast cancer has been a primary threat to women's health.^1^ Among cytotoxic chemotherapeutic agents for advanced breast cancer patients, prior to anthracycline therapy, is gemcitabine (GEM).^2^ However, there are still some drawbacks of GEM treatment that compromise its applications.^3^ For example, GEM lacks selectivity towards cancer tissues and enters cancer and healthy cells indiscriminately, which led to severe side effects and narrow therapeutic windows.^3^ In addition, it was reported that approximately 90% of GEM would be rapidly decomposed with a short half-life of 32 min in blood circulation due to the deamination to produce an inactive 2′,2′-difluorodeoxyuridine.^2^ Furthermore, cancer cells tend to become drug resistant after prolonged treatment with GEM.^4^

Combination chemotherapy is an effective strategy for alleviating the above problems.^5–8^ In comparison with conventional single-agent treatment, combination therapy is capable of promoting synergistic effects of different drugs, enhancing therapeutic selectivity and overcoming multidrug resistance *via* distinct mechanisms of actions.^9–11^ However, combination chemotherapy also has its own drawbacks due to different pharmacokinetic properties of the drugs, which increases the difficulty to obtain the optimal dose and further causes more adverse side effects.^12,13^

To address this dilemma, nanocarrier-based drug delivery systems (nano-DDS) have drawn more and more attentions for promoting therapeutic efficacy and overcoming the pharmacokinetic limitation of anti-cancer drugs by improving the balance between their efficacy and toxicity.^14^ Especially polymer–drug conjugate, also known as polymeric prodrug, is the most investigated carriers and has demonstrated significant potentials in nanomedicine for their advanced drug delivery to the targets, by improving drug bioavailability, balancing the pharmacokinetics of hydrophilic and hydrophobic drugs, allowing the tumor-specific activation of drugs.^15–19^ Therefore, polymeric prodrugs provide a potential platform for effective anticancer drug delivery by synchronizing the advantages of polymeric prodrug and co-delivery systems, and are capable of achieving synergistic therapeutic effects through simultaneous multi-drug accumulation.^20–23^ Nevertheless, developing such a drug delivery system still faces technical challenges such as lacks of specific drug target and rapid intracellular drug release at the target site.^24^

Therefore, introduction of different biological ligands or antibodies into novel drug delivery system would be beneficial for selective delivery of chemotherapeutics to tumor cells.^25^ Folic acid (FA) is regarded as an important and active targeting agent, which shows a strong affinity for specific receptors that are uniquely overexpressed in tumor cells.^26,27^ Thus, introduction of FA onto polymer–drug conjugate would increase the drug accumulation at the tumor sites and promote cancer cell uptake *via* a folate receptor-mediated endocytosis, thereby improving drug bioavailability and enhancing the therapeutic of cancer treatments.^28,29^

**Fig. 1:**
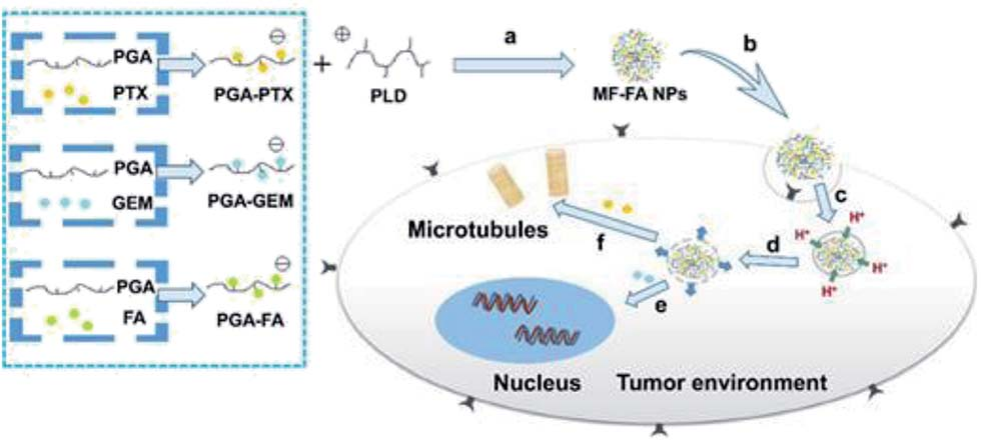
Schematic illustration of the preparation of MF-FA NPs and the activated release behavior of PTX and GEM in tumor cells. (a) Self-assembly through electrostatic interaction. (b) Endocytosis. (c) Taken up by lysosomes. (d) Endosomal escape by proton influx. (e) GEM inhibits DNA replication. (f) PTX breaks down microtubules.

In this manuscript, we developed a folate targeting multi-functionalized nanocarrier delivery system for co-delivery of hydrophilic chemotherapeutic drug GEM and hydrophobic PTX for the breast cancer treatment. The novel polymeric prodrug nanoparticles were constructed through electrostatic interactions using synthesized poly glutamic acid (PGA) conjugated GEM (PGA–GEM), PGA conjugated PTX (PGA–PTX), PGA conjugated FA (PGA–FA) and poly-lysine dendrimer (PLD/P-6). The two anti-cancer drugs and targeting ligand FA were all coupled to the polymer backbone of PGA through hydrolysable ester bond linkers. PLD was introduced as a cationic and biodegradable dendrimer to promote the self-assembly of polymeric prodrug and further increase the stability of multi-functionalized nanoparticles. In addition, PLD would also facilitate the drugs to escape from lysosome *via* the proton sponge effect to increase therapeutic efficacy.^30^ As illustrated in Fig. 1, the nanoparticles would preferentially accumulate at tumor tissue by passive targeting effect and enter tumor cells *via* folate receptor mediated endocytosis. Once the polymer enters the cells, ester bond of the polymer would be rapidly cleaved in response to the acidified endosomes and GEM and PTX would be sufficiently released, thereby leading to synergistic anticancer effects.

**Fig. 2:**
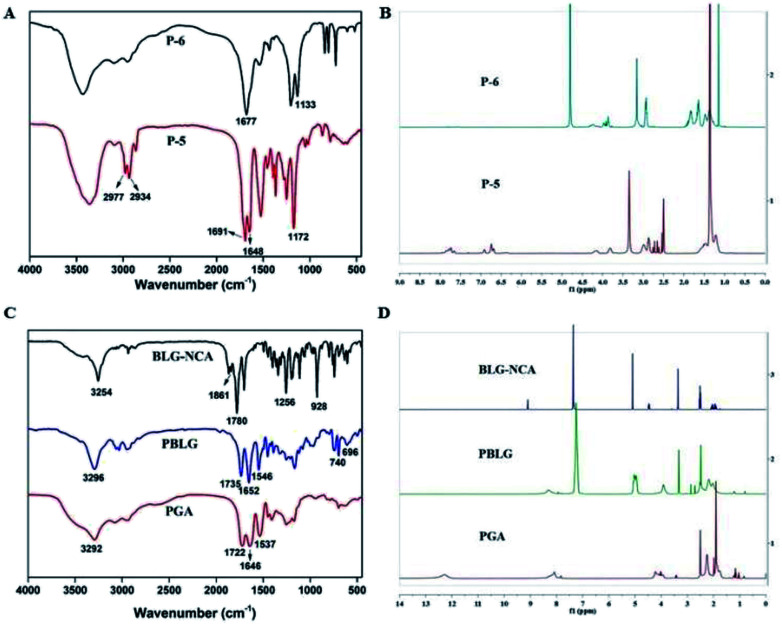
Characterization of the compounds. (A) FT-IR spectra of P-5 and P-6. (B) ^1^H NMR spectra of P-5 and P-6. (C) FT-IR spectra of BLG-NCA, PBLG and PGA. (D) ^1^H NMR spectra of BLG-NCA, PBLG and PGA.

## 2. Materials and methods

### 2.1 Materials

1-Hydroxybenzotriazole (HOBT) was purchased from Accela ChemBio Co., Ltd (Shanghai, China). 1-(3-Dimethylaminopropyl)-3-ethylcarbodiimide hydrochloride (EDCI), folic acid hydrate (FA), *N*-hydroxysuccinimide (NHS), 1,6-diaminohexane, l-glutamic acid (Glu), ethyldiisopropylamine (DIPEA) and trifluoroacetic acid (TFA) were obtained from Energy Chemical Technology Co., Ltd (Shanghai, China). *Tert*-butyl methyl ether was purchased from Shanghai Titan Scientific Co., Ltd (Shanghai, China). Boc-Lys, paclitaxel (PTX) and gemcitabine (GEM) were purchased from Bide Pharmatech Ltd (Shanghai, China).

### 2.2 Synthesis of polymer–drug conjugates

#### 2.2.1 Synthesis of poly-lysine dendrimer (PLD/P-6)

The synthetic route of the poly-lysine dendrimer (PLD/P-6) was shown in Fig. S1.† Boc-Lys (1.00 g, 1.89 mmol) and HOBT (0.64 g, 4.74 mmol) were dissolved in DCM (50 mL). The mixture was stirred at −10 °C for 10 min, followed by adding EDCI (0.91 g, 4.74 mmol) and 1,6-hexanediamine (0.11 g, 0.95 mmol) into the system and stirred for another 30 min. Subsequently, DIPEA (0.73 g, 5.67 mmol) was added and the reaction was allowed to proceed for overnight under N_2_ atmosphere. Afterwards, the system was washed with HCl solution (20 mL × 2), 5% NaHCO_3_ (20 mL × 2), saturated sodium chloride solution and further dried over anhydrous Na_2_SO_4_. Then the solution was concentrated under vacuum to give compound P-1. To remove the protecting group Boc, P-1 (0.85 g, 1.10 mmol) was dissolved in 5 mL DCM, followed by adding of trifluoroacetic acid (TFA) (1.23 mL, 16.50 mmol). The mixture was reacted for 3 h, and the organic solvent was evaporated in vacuum and the residue was washed with methyl *tert*-butyl ether to give product P-2. The stepwise growth of Lys was repeated to yield P-5, the protecting group Boc of which was removed to give PLD/P-6. P-5: ^1^H NMR (400 MHz, DMSO-d_6_) *δ* 7.98–7.59 (–CONH), 4.16–3.82 (–CH), 2.90 (–CH_2_), 1.48 (–CH_2_), 1.36 (–CH_3_), 1.33–1.28 (–CH_2_), 1.22 (–CH_2_). FTIR peak (cm^−1^): 2997, 2934, 1691, 1648, 1172. P-6: ^1^H NMR (400 MHz, D_2_O) *δ* 4.04–3.82 (–CH), 3.31–3.02 (–CH_2_), 3.00–2.84 (–CH_2_), 2.05–1.69 (–CH_2_), 1.66–1.43 (–CH_2_), 1.40–1.11 (–CH_2_). FTIR peak (cm^−1^): 1677, 1133.

The average molecular weight of P-6 was determined by gel permeation chromatography (PL GPC 50). The dissolution solvent was water and mobile phase was composed of 0.2 M NaNO_2_. GPC was performed on a column (2 × PL aquagel-OH 308 μm, 7.5 × 300 mm) at a 1.0 mL min^−1^ flow rate with a refractive index detector (sample concentration: 0.10 mg mL^−1^, injection volume: 100.0 μL). The average molecular weight (2403 Da) and polydispersity index (1.08) were calculated from the GPC retention time (17.33 min, *y* = 11.104568 − 0.445890*x*).

#### 2.2.2 Synthesis of poly glutamic acid (PGA)

The synthetic route of the poly glutamic acid (PGA) was shown in Fig. S2.† In brief, Glu (Z) (5.00 g, 211.00 mmol) was dissolved in anhydrous THF (100 mL), then triphosgen (6.25 g, 21.06 mmol) was added and the system took place at 50 °C for 3 h under N_2_ atmosphere. After removing the remaining organic solvent in vacuum, the residue was washed with cold *n*-hexane to obtain purified BLG-NCA. The average molecular weight was determined by gel permeation chromatography (PL GPC 50) (sample concentration: 0.10 mg mL^−1^, injection volume: 100.0 μL). The average molecular weight (6425 Da) and polydispersity index (1.07) were calculated from the GPC retention time (17.13 min, *y* = 11.104568 − 0.445890*x*). ^1^H NMR (400 MHz, DMSO-d_6_) *δ* 7.37–7.30 (–Ph), 5.08 (–COOCH_2_), 4.47 (–CHNH), 2.61–2.44 (–CH_2_), 2.08–1.85 (–CH_2_). FTIR peak (cm^−1^): 3254, 1861, 1780, 928.

For synthesis of PBLG, BLG-NCA (4.97 g, 18.89 mmol) and *N*-hexylamine (25.48 mg, 0.25 mmol) were dissolved in DMF (25 mL) in a 100 mL round-bottom eggplant flask. The mixture reaction was reacted at 35 °C for 72 h. Subsequently, the reaction mixture was poured into 500 mL of cold methyl *tert*-butyl ether, and the precipitate was dried in vacuum and white powder PBLG was obtained. ^1^H NMR (400 MHz, DMSO-d_6_) *δ* 7.94 (–CONH), 7.29 (–Ph), 5.20–4.79 (–CH_2_), 3.91 (–CH), 3.04 (–CH_2_), 2.18 (–CH_2_), 2.03 (–CH_2_), 1.40 (–CH_2_), 1.21 (–CH_2_), 0.80 (–CH_3_). FTIR peak (cm^−1^): 3296, 1652, 1546, 740, 696.

In order to prepare PGA, PBLG (5.00 g, 22.62 mmol) was dissolved in MeOH (15 mL). Then 3.6 g of NaOH (90.00 mmol) was dissolved in H_2_O (2 mL) and injected into the mixture. The mixture was stirred for 5 h at room temperature. Then 30 mL of EtOH was poured into the system and centrifuged at 6000 rpm min^−1^ for 10 min to separate precipitate, which was further washed for several times until the pH was 7–8. Subsequently, the dried precipitate was dissolved in 5 mL of HCl/EA solution. After 10 min of stirring, the remaining solvent was removed to obtain PGA. ^1^H NMR (400 MHz, DMSO-d_6_) *δ* 12.28 (–COOH), 8.13 (–CONH), 4.27–3.82 (–CH), 3.43 (–CH_2_), 2.25 (–CH_2_), 2.00–1.68 (–CH_2_), 1.37 (–CH_2_), 1.23 (–CH_2_), 0.84 (–CH_3_). FTIR peak (cm^−1^): 3292, 1722, 1646, 1537.

#### 2.2.3 Preparation of PGA–PTX and PGA–GEM

**Fig. 3:**
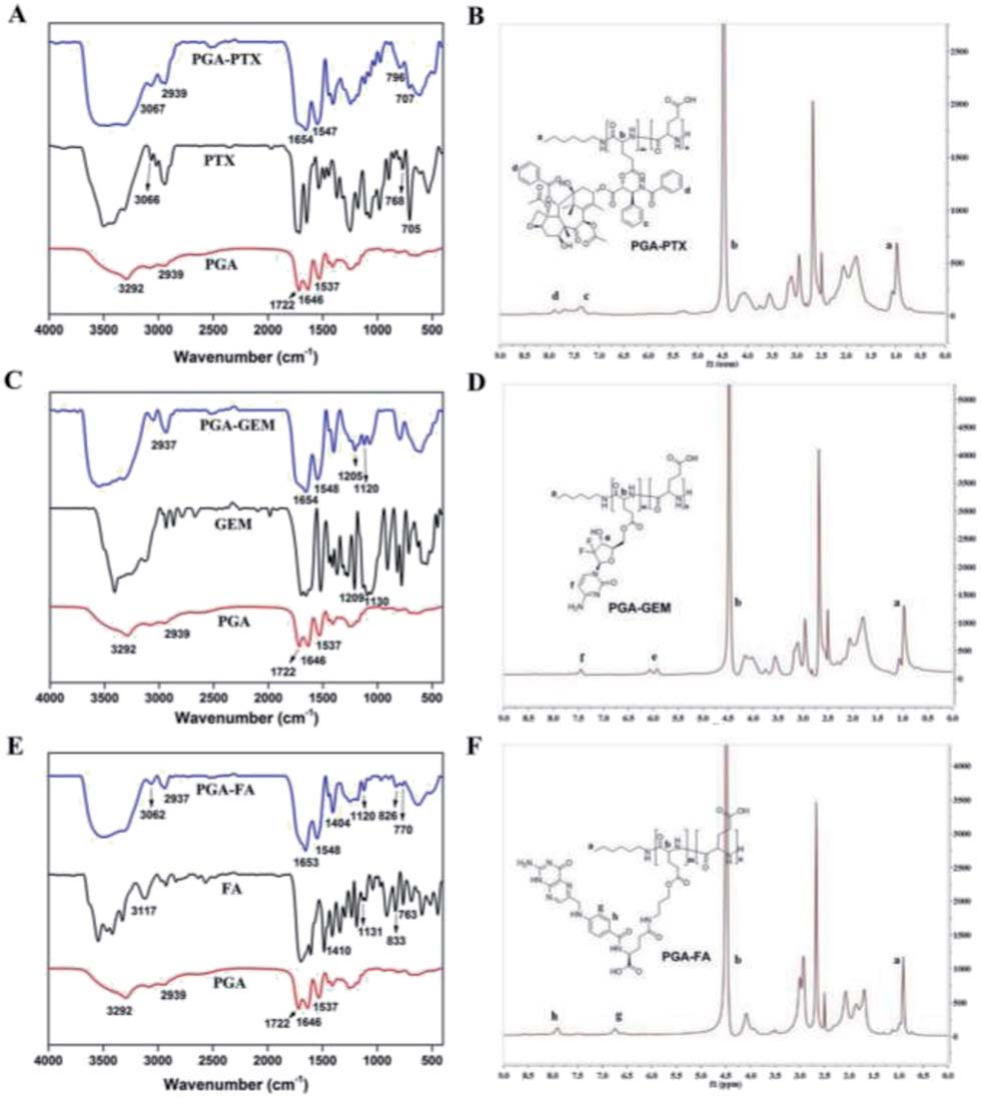
FT-IR spectra and ^1^H NMR spectra of PGA–PTX, PGA–GEM and PGA–FA.

PGA (0.40 g, 3.30 mmol) was dissolved in 5 mL of DMSO, followed by adding EDCI (0.30 g, 1.56 mmol) and PTX (60.00 mg, 0.07 mmol) to the mixture. The system was stirred at room temperature overnight and the product was dialyzed against deionized water with cellulose tubing (MWCO: 1000 Da), followed by lyophilization to obtain PGA–PTX. ^1^H NMR (400 MHz, DMSO-*d*_6_) *δ* 7.89–7.68 (–Ph-d), 7.33 (–Ph-c), 4.49 (–COCHNH-b), 1.02 (–CH_3_-a) (Fig. 3B). FTIR peak (cm^−1^): 3066, 768, 705.

For synthesis of PGA–GEM, PTX was replaced by GEM, and the preparation was similar to that of PGA–PTX. ^1^H NMR (400 MHz, DMSO-*d*_6_) *δ* 7.45 (–Ph-f), 5.99 (–CF_2_CHO-e), 4.49 (–COCHNH-b), 0.97 (–CH_3_-a) (Fig. 3D). FTIR peak (cm^−1^): 1209, 1130.

#### 2.2.4 Synthesis of PGA–FA

The synthetic route of the modified PGA with FA was displayed in Fig. S3.† Generally, FA (1.07 g, 2.41 mmol) was dispersed in 20 mL of DMSO, then NHS (277.00 mg, 2.41 mmol) and EDCI (694.00 mg, 3.60 mmol) were added and the resulting solution was stirred for 1 h at room temperature. Afterwards, 0.5 mL of Et_3_N was added into the solution and stirred for another 36 h in the dark. The reaction system was poured into a mixture of methyl *tert*-butyl ether: acetone (40 mL; 7 : 3, v/v), and the precipitate was dried in vacuum and FA-NHS was obtained. Subsequently, FA-NHS (200.00 mg, 0.37 mmol) was dissolved in 8 mL of DMSO, then Et_3_N (151.90 mg, 0.56 mmol) and 3-aminopropanol (27.90 mg, 0.37 mmol) were added respectively. The mixture was stirred for 24 h in the dark at room temperature. The system was poured into a mixture of methyl *tert*-butyl ether : acetone (20 mL; 7 : 3, v/v), and the precipitate was dried in vacuum and light yellow product FA-OH was obtained. For the preparation of PGA–FA, PGA (200.00 mg, 1.65 mmol) was dispersed in 4 mL of DMSO, followed by adding EDCI (316.86 mg, 1.65 mmol) and DMAP (201.95 mg, 1.65 mmol) respectively. The mixture was stirred for 30 min, then FA-OH (66.00 mg, 0.13 mmol) was added and further stirred for 24 h. The mixture was then dialyzed against deionized water with cellulose tubing (MWCO: 1000 Da), followed by lyophilization to obtain PGA–FA. The ^1^H NMR and FTIR spectra of PGA–FA were as follows: ^1^H NMR (400 MHz, DMSO-*d*_6_) *δ* 7.48 (–Ph-h), 6.66 (–Ph-g), 4.49 (–COCHNH-b), 0.94 (–CH_3_-a) (Fig. 3F). FTIR peak (cm^−1^): 3117, 1410, 1131, 833, 763.

### 2.3 Preparation and characterization of functionalized nanoparticles

Multi-functional FA targeting nanoparticles (MF-FA NPs) were prepared by electrostatic interaction between positively charged P-6 and negatively charged PGA–PTX, PGA–GEM and PGA–FA. In brief, 5.00 mg of PGA–PTX, PGA–GEM and PGA–FA was dissolved in 10 mL of distilled water, respectively. Then 300 μL of each sample was fully mixed by vortex. Subsequently, different ratio of P-6 solution was added to the above mixed solution, and further were incubated at 4 °C for 3 h to obtain the nanoparticles.

Particle size distribution and zeta potential were measured using a Malvern Zetasizer 3000 system (Malvern Instruments Ltd., UK). In order to observe the morphology of NPs, the samples were obtained by depositing the nanoparticles solution onto double carbon coated copper grid and drying at room temperature. Transmission electron microscopy (TEM) was applied to observe morphology of nanoparticle samples.

### 2.4 Determination of drug loading

Different concentrations of PTX, GEM and FA were prepared and measured by UV-Vis spectrophotometer to get standard curve. The absorbance of PTX, GEM and FA which were encapsulated in PGA–PTX, PGA–GEM and PGA–FA were determined by UV-Vis spectrophotometer at 230 nm, 269 nm and 280 nm, respectively. The drug concentration of PGA–PTX, PGA–GEM and PGA–FA were calculated by the standard curve. The drug loading content was calculated by the following equation:
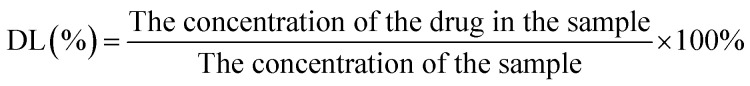


### 2.5 *In vitro* drug release of PTX and GEM

The drug release profiles of PTX and GEM were investigated by the dialysis bag diffusion technique. Briefly, 2 mL of MF-FA NPs solution (1.5 mg PTX and 1.5 mg GEM) was placed in a dialysis bag (MWCO = 3500 Da) and immersed in a 30 mL of PBS buffer solution containing 0.5% Tween 80 at pH 5.5 and pH 7.4. The released solution was kept at 37 °C with shaking at 100 rpm. At predetermined times, 1 mL of samples were withdrawn and replaced with an equivalent volume of fresh medium. The concentration of PTX and GEM was analyzed by Ultra-Performance Liquid Chromatography (UPLC). The mobile phase was composed of DPBS/ACN (50 : 50, v/v) with a flow rate of 1 mL min^−1^. A C18 column (BDS Hypersil C18 5 μm, 4.6 × 150 mm column) was used in UPLC analysis where PTX was detected at 230 nm and GEM was detected at 269 nm (PTX: 0.93 min, *y* = 0.2191*x* − 0.2455, *R*^2^ = 0.998; GEM: 5.94 min, *y* = 0.2883*x* − 0.0252, *R*^2^ = 0.998), respectively.

### 2.6 Stability of nanoparticles

The stability of MF-FA NPs was evaluated by monitoring changes in particle size, PDI and zeta potential in distilled water. MF-FA NPs solution (0.5 mg mL^−1^) was kept at room temperature for 96 h, and at prearranged intervals the parameters were determined.

### 2.7 Hemolysis study

Hemolysis study was investigated using Micro free hemoglobin kit (Nanjing Jian Cheng Bioengineering Institute). Firstly, fresh whole blood was collected from healthy mice, and was diluted in normal saline to separate red blood cell (RBC) from the whole blood through centrifugation at 2000 rpm for 10 minutes at 4 °C, which was then washed three times with normal saline and further diluted with saline to obtain a 2% RBC suspension (v/v). Subsequently, the red blood cell suspension was treated with MF-FA NPs and MF NPs, distilled water and saline, respectively.^31^ They were incubated at 37 °C for 1 h and centrifuged at 2000 rpm for 10 minutes to obtain the supernatant. Standard operating procedures of the assay by the kit manufacturer were followed and the data was analyzed at 510 nm by UV-Vis spectrophotometer (UV-2550, Kyoto, Japan).

### 2.8 Study of antiproliferative ability

The antiproliferative ability of drug-loaded nanoparticles and blank nanoparticles was evaluated by the Cell Counting Kit-8 (CCK-8) assay. The breast cancer cell line 4T1 was provided by Shanghai Cell Institute (Shanghai, China). The cells were cultured in RPMI 1640 with 10% fetal bovine serum (FBS), 100 U mL^−1^ penicillin and 100 U mL^−1^ streptomycin, and incubated at 37 °C in 5% CO_2_. Then cells were seeded in 96-well plates at a density of 5 × 10^3^ cells per well and cultured for 24 h. The cellular uptake of drugs *via* receptor-mediated endocytosis occurred in a short time period. Then serial dilutions of GEM and PTX were maintained in all cases: MF-FA NPs, MF NPs and pure drug (PTX : GEM = 1 : 1). After further incubation for up to 72 h, 10 μL of CCK-8 solution was added to each well and incubated for 3 h. Cell viability was calculated by a microplate reader (BMG CLARIO star, German) at 450 nm.

### 2.9 *In vivo* efficacy study

BALB/c mice (20 ± 2 g, 6–8 weeks old, female) were obtained from Shanghai SIPPE-BK laboratory animal Co., Ltd (Shanghai, China) and acclimatized for 7 days after arrival. The mice were housed in cages with free access to food and water. All animal procedures were performed in accordance with the Guidelines for Care and Use of Laboratory Animals of Nanjing Normal University, and the animal use protocol was approved by the ethics committee of Nanjing Normal University. Tumor-bearing BALB/c mice were prepared by injection of 1 × 10^6^ 4T1 cells per mouse in the left armpit. When the tumor volume reached approximately 80 mm^3^, mice were randomly divided into five groups with three mice in each group and intravenously administered with saline, pure drug (PD), MF-FA NPs, MF NPs, respectively. Saline was used as a negative control, and pure drug as a positive control. The group of pure drug exhibited serious toxicity over the concentration of 5 mg kg^−1^. Nano-groups showed good biocompatibility at the concentration of 10 mg kg^−1^ due to slow release of drugs. So nano-groups and pure drug group were injected *via* the tail vein at a dose of 10 mg kg^−1^ and 5 mg kg^−1^ respectively every other day.

All mice were tagged and the tumor volume was calculated according to the formula of (*a* × *b*^2^)/2, where “*a*” was the major axis and “*b*” was the minor axis. At the same time, the body weight of the mice was also monitored. Treatment proceeded for 21 days. After day 21, the mice were sacrificed and tumors were excised.

### 2.10 Statistical analysis

At least three replicates were used in the study, and data were expressed as mean ± standard deviation (SD). Statistical evaluation between different groups was analyzed by Student's *t*-test or one-way ANOVA. *P* < 0.05 indicated statistical significance in experiments.

## 3. Results and discussion

### 3.1 Synthesis and characterization of polymer–drug conjugates

#### 3.1.1 Synthesis and characterization of P-6

P-6 was formed by the gradual reaction of 1,6-hexanediamine as a core under the homogeneous conditions. Firstly, 1,6-hexanediamine reacted with Boc-Lys under amide condensation and P-1 was obtained. Then the removal of Boc group of P-1 using trifluoroacetic acid (TFA) gave P-2. The stepwise growth of Lys was repeated to yield P-6. The structure of compounds were confirmed by FT-IR (Bruker, VERTEX 80V, Germany) and ^1^H-NMR spectra (Varian INOVA 400 MHZ nuclear magnetic resonance instrument).

In the IR spectrum of P-5 (Fig. 2A), the stretching vibrations of C–H on *tert*-butyl were observed at 2977 cm^−1^ and 2934 cm^−1^. Peaks at 1691 cm^−1^ and 1648 cm^−1^ could be attributed to the C=O on the Boc group. In the IR spectrum of P-6, the intensity of four peaks above were significantly reduced and the peak at 1133 cm^−1^ corresponded to stretching vibration of primary amine C–N. Additionally, in the ^1^H NMR spectra of P-5 and P-6 (Fig. 2B), the disappearance of hydrogen of P-6 at 1.36 ppm (–CH_3_) indicated the successful removal of the Boc group. All the data confirmed the structure of P-6.

#### 3.1.2 Synthesis and characterization of PGA

For synthesis of PGA, BLG-NCA was prepared using Glu (Z) with triphosgen. Then, PBLG was polymerized by ring opening from BLG-NCA using *N*-hexylamine as a nucleophilic initiator. PBLG reacted with NaOH to remove benzyl and PGA was obtained.

As shown in Fig. 2C, in the IR spectrum of BLG-NCA, the observed absorbance bands at 1861 cm^−1^, 1780 cm^−1^ and 1256 cm^−1^ were characterized for the stretching vibrations of the two C=O. The peak at 928 cm^−1^ corresponded to stretching vibrations of O=C–O–C=O. The results indicated successful synthesis of BLG-NCA. In the IR spectrum of PBLG, the disappearance of characteristic absorption peaks of BLG-NCA (1861 cm^−1^, 1780 cm^−1^, 1256 cm^−1^ and 928 cm^−1^) indicated that the BLG-NCA monomer was completely polymerized. The peak at 3296 cm^−1^ and 1652 cm^−1^ were the stretching vibration absorption peak of amino group and carbonyl in the amide respectively. The peak at 1546 cm^−1^ was assigned to bending vibration of N–H in amide and C–N stretching vibration, which indicated that the BLG-NCA monomer was bonded with peptide to form PBLG. The peaks at 740 cm^−1^ and 696 cm^−1^ were attributed to the deformation vibration of benzene ring. In the IR spectrum of PGA, the disappearance of 740 cm^−1^ and 696 cm^−1^ indicated that benzyl group was removed. Therefore, the results indicated successful synthesis of PGA.

Structures of BLG-NCA, PBLG and PGA were further confirmed by ^1^H NMR, which were showed in Fig. 2D. For the BLG-NCA, 2.0 ppm and 2.45 ppm were related to –CH_2_ and 4.47 ppm was related to –CHNH. Signal at 5.08 ppm was methylene peak of –CH_2_Ph and 7.3 ppm was the characteristic of –Ph peak. The characteristic of –Ph peak at 7.29 ppm and methylene peak of benzyl at 5.2 ppm can be found in the ^1^H NMR spectrum of PBLG. In PGA, both the disappearance of these two peaks and the appearance peak of –COOH in 12.28 ppm verified its structure. The degree of polymerization (50) of PBLG was quantified from the peak integration ratio of hexylamine at 0.80 ppm (–CH_3_) to the α-carbon proton at 3.91 ppm (–CH) of PBLG. Therefore, the results indicated successful synthesis of PGA.

#### 3.1.3 Synthesis and characterization of PGA–PTX, PGA–GEM and PGA–FA

PGA reacted with GEM and PTX through dehydration condensation. Additionally, FA-OH was synthesized by 3-aminopropanol and the active hydroxylic group of FA. The carboxyl groups of PGA were conjugated with the hydroxyl group of FA-OH *via* esterification and gave the PGA–FA.

As shown in Fig. 3A, in FT-IR spectra of PTX, the observed absorbance bands at 705 cm^−1^, 768 cm^−1^ and 3066 cm^−1^ were characteristics for the bending vibration of C–H on benzene ring. The bending vibration at 1646 cm^−1^ and 1537 cm^−1^ was attributed to the C=O and N–H in PGA, respectively. The observed absorbance bands at 3292 cm^−1^ and 1722 cm^−1^ were characteristics for the stretching vibration of –COOH in PGA. The peak at 2939 cm^−1^ was attributed to the stretch absorption of –CH_2_ in PGA and PGA–PTX. The appearance of the above characteristic peaks in PGA–PTX proved the structure of PGA–PTX. IR spectrum of PGA–GEM was shown in Fig. 3C, the peak at 1205 cm^−1^ was attributed to the stretching vibration of C–F and the peak at 1120 cm^−1^ was attributed to the stretching vibration of C–O–C. The appearance of the above characteristic peaks in PGA–PTX proved the structure of PGA–PTX. As shown in Fig. 3E, the observed absorbance bands of FA at 3117 cm^−1^, 833 cm^−1^ and 763 cm^−1^ were characteristic for the bending vibration of C–H on benzene ring. The stretching vibration at 1410 cm^−1^ and 1131 cm^−1^ were attributed to the C–N in FA. The peaks (3062, 2937, 1653, 1548, 1404, 1120, 826, 770 cm^−1^) in PGA–FA indicated successful synthesis of PGA–FA. Therefore, the results confirmed successful synthesis of PGA–PTX, PGA–GEM and PGA–FA.

In ^1^H NMR spectra of PGA–PTX (Fig. 3B), the peaks of PGA or PTX appeared at 1.02 ppm (–CH_3_-a), 4.49 ppm (–COCHNH-b), 7.33 ppm (–Ph-c) and 7.89–7.68 ppm (–Ph-d). In ^1^H NMR spectra of PGA–GEM (Fig. 3D), the peaks of PGA or GEM appeared at 7.45 ppm (–Ph-f), 5.99 ppm (–CF_2_CHO-e), 4.49 ppm (–COCHNH-b) and 0.97 ppm (–CH_3_-a). In ^1^H NMR spectra of PGA–FA (Fig. 3F), the peaks of PGA or FA appeared at 7.48 ppm (–Ph-h), 6.66 ppm (–Ph-g), 4.49 ppm (–COCHNH-b), 0.94 ppm (–CH_3_-a). Consequently, PTX, GEM and FA were successfully conjugated with the carboxylic acid of PGA.

### 3.2 Preparation and characterization of functionalized nanoparticles

The particle size and zeta potential of different proportions were shown in Fig. S4.† The result indicated that the ratio of 1.5 : 1 exhibited the smallest particle size and suitable zeta potential compared with other ratios. So we chose the ratio of 1.5 : 1 to carry out the studies.

**Fig. 4:**
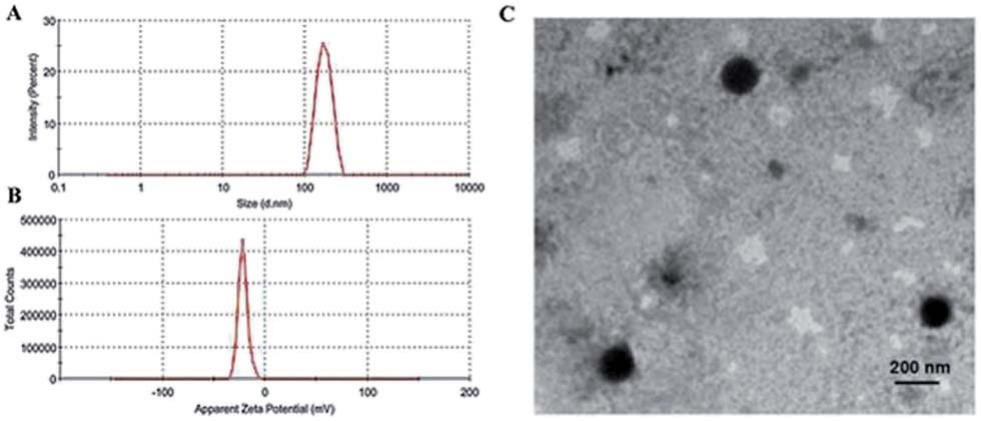
Morphology of MF-FA NPs. (A) Size distributions. (B) Zeta potential distributions. (C) TEM of MF-FA NPs.

As summarized in Fig. 4A, the average particle size of MF-FA NPs was about 170 nm, with a PDI of 0.232. In addition, the zeta potential of the MF-FA NPs was −21.60 mV (Fig. 4B). The particle size of MF-FA NPs observed by TEM was about 160 nm (Fig. 4C), which was smaller than the results determined by DLS. The particle size determined by DLS represented the hydrodynamic diameter, whereas the results obtained by TEM were the collapsed nanoparticles after water evaporation.^32^ Moreover, the drug loading (DL) was measured to further evaluate the nanoparticles. The DL of PGA–PTX, PGA–GEM and PGA–FA were 18.6%, 19.56% and 19.4% respectively, and the experimental results were as expected as theory.

### 3.3 *In vitro* drug release of PTX and GEM

**Fig. 5:**
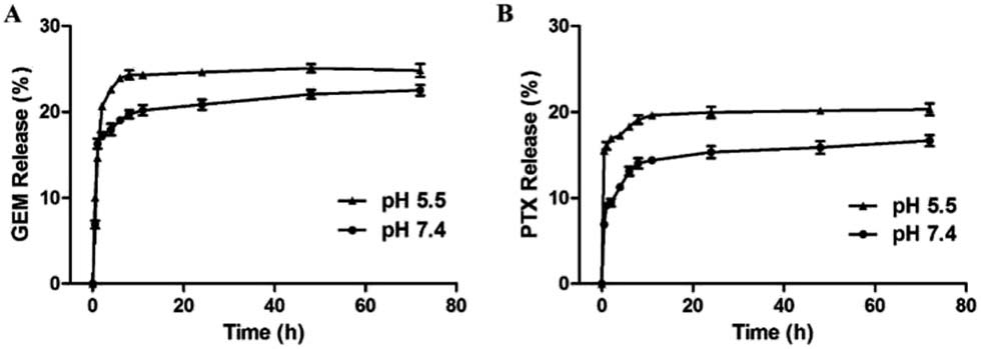
Release profiles of GEM (A) and PTX (B) from MF-FA NPs in PBS (pH 5.5 and pH 7.4, respectively) containing 0.5% (v/v) of Tween 80.

Effective anticancer carriers should provide targeting drug release and accumulation at tumor site and subsequently lead to satisfactory therapeutic results. Therefore, *in vitro* drug release profiles for MF-FA NPs were investigated in different environments (pH 5.5 and 7.4), as shown in Fig. 5. The cumulative PTX and GEM release amount at pH 5.5 were remarkably higher than pH 7.4, indicating that the release of drug was affected by pH condition, which could be attributed to the easy cleavage of ester bonds in acidic environments. In addition, the nanoparticles presented a rapid drug release in the first 12 hours, especially in pH 5.5. According to recent reports, for cancer treatment, it would be more desirable to achieve rapid and sufficient drug release once the carriers reach at the tumor site to obtain better therapeutic efficacy. Hence, the above results illustrated that MF-FA NPs exhibited a typical pH dependent and rapid drug release pattern, which would facilitate selective drug release within tumor cells.

### 3.4 Stability of nanoparticles

**Fig. 6:**
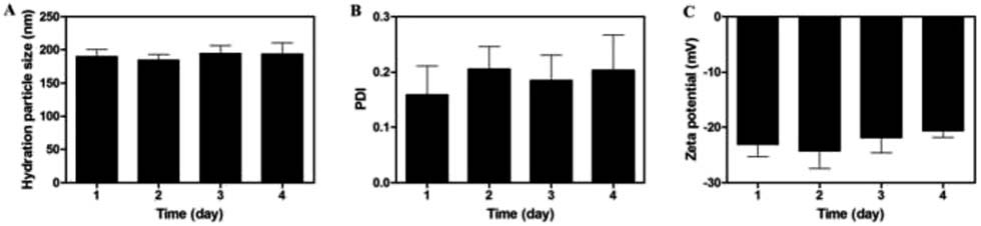
Hydration particle size (A), PDI (B) and zeta potential (C) of MF-FA NPs in four days at room temperature.

The stability of nanoparticles is crucial to their applications, such as long-term storage stability, prolonged biological activities at tumor site and circulation in body. As depicted in Fig. 6, no significant changes were detected in terms of particle size, zeta potential and PDI within 96 h, indicating that the nanoparticles would remain stable at room temperature at least for up to four days.

### 3.5 Hemolysis study

**Fig. 7:**
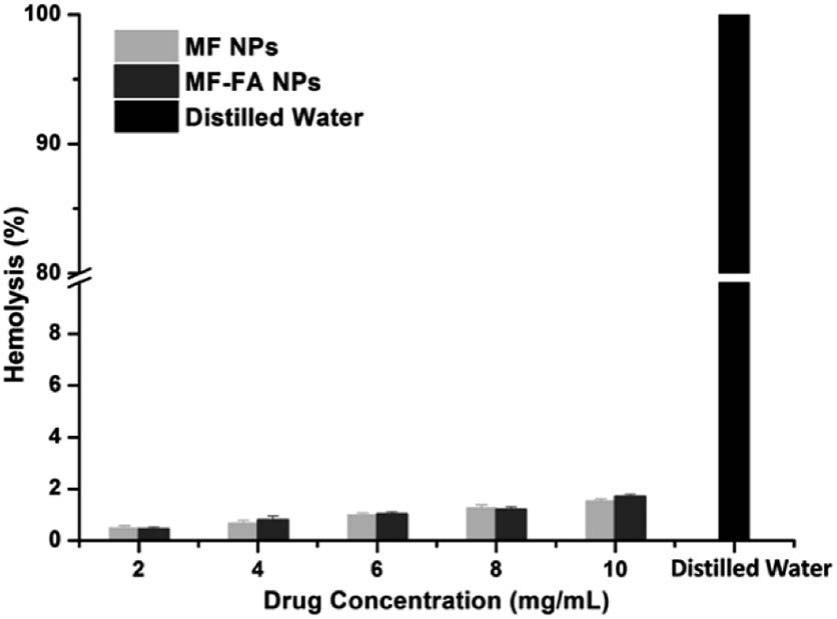
Hemolysis of red blood cells after incubation with MF NPs, MF-FA NPs and distilled water.

The hemolysis study was carried out to evaluate the biocompatibility of nanoparticles. A hemolytic percentage less than 2% is considered as non-hemolytic and 2–5% are slightly hemolytic and more than 5% is hemolytic, according to ASTM F756-13 standard for nanomaterials. Distilled water caused significant damage of red blood cells (Fig. 7), and saline did not induce substantial hemolysis. In contrast, the hemolytic percentage of MF-FA NPs and MF NPs were all less than 2% when both nanoparticles were tested from 2 to 10 mg mL^−1^ (Fig. 7), which indicated good safety for the nanomaterials.

### 3.6 Study of antiproliferative ability

**Fig. 8:**
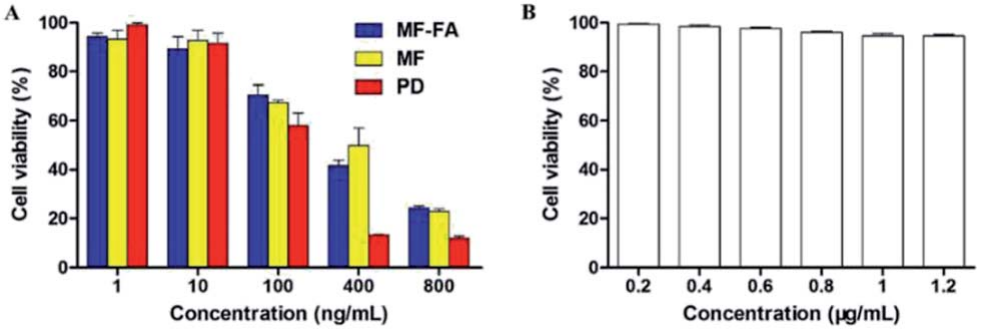
Cell proliferation assessments using the CCK-8 assay. (A) The proliferation inhibition of MF-FA NPs, MF NPs and pure drug to 4T1 cells at 72 h. (B) The proliferation inhibition of blank NPs to 4T1 cells at 72 h.

The antiproliferative ability of drug-loaded MF-FA NPs, MF NPs and pure drug (PTX : GEM = 1 : 1) were evaluated using a CCK-8 assay on breast cancer cells 4T1. As shown in Fig. 8A, FA modified MF-FA NPs exhibited a better proliferation inhibition to 4T1 cells with the concentration of 10 and 400 ng mL^−1^ than MF NPs after incubation for 72 h. The enhanced proliferation inhibition to 4T1 cells was owing to the folic acid specific targeting in the nanoparticles. However, other concentration of NPs exhibited no significant differences between MF-FA NPs and MF NPs perhaps due to the experimental errors. The results demonstrated that FA enhanced cellular uptake of the nanoparticles.

Besides, pure drug showed higher inhibition against 4T1 cells than the two nanoparticles due to the direct transportation into cells. While for the MF-FA NPs and MF NPs, the drugs were slowly released and the absorption by cells was delayed as previously reported in the vitro drug release study.^33^

In low concentration (1 ng mL^−1^), nanoparticles were enriched around cancer cells and sustained release drugs. Pure drug was dispersed in the culture solution and led to the lower inhibition against 4T1 cells than the two NPs. The results in Fig. 8B showed that blank carriers did not exhibit proliferation inhibition to 4T1 cells, which further illustrated that drug-loaded NPs had high biocompatibility.

### 3.7 *In vivo* antitumor efficacy

**Fig. 9:**
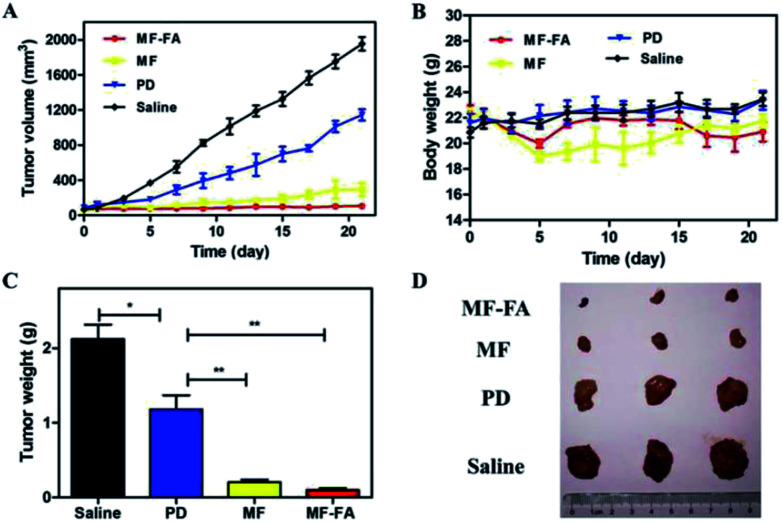
*In vivo* anti-tumor effects of breast cancer 4T1 cell-induced BALB/c mice. (A) Tumor growth curves. (B) Body weights of the mice. (C) Average tumor weight after the study. (D) Images of tumor tissues. Data were shown as mean ± SD, (*n* = 3). Significant difference from control: *P < 0.05, **P < 0.01, ***P < 0.001.

The antitumor efficacies of MF-FA NPs, MF NPs and pure drug were evaluated in breast tumor bearing mice. As shown in Fig. 9A, in the control group, tumors quickly and continuously grew and the average tumor volume on day 21 was up to 1900 mm^3^ approximately. In other three groups, the curves of tumor volume were increased more slowly than the control group in 21 days. Compare with the pure drug group, the MF NPs group had a significant anti-tumor effect. Furthermore, the antitumor activity of FA-loaded NPs (MF-FA NPs) was more effective than FA-free NPs (MF NPs) due to the high expression of FA receptor in cancer cells. The results indicated that the targeting moiety FA of MF-NPs was able to effectively deliver PTX and GEM. In addition, the body weight of mice did not show significant loss in the duration of the study (Fig. 9B), indicating satisfactory safety of the testing nanoparticles. The tumor weights of NPs groups were significantly reduced compared with saline group (Fig. 9C and D) and the average weights of tumor in MF-FA NPs, MF NPs, PD and control groups were 0.09 g, 0.20 g, 1.18 g and 2.12 g, respectively. There were significant differences (*p* < 0.001) between saline and NPs groups.

Consequently, these results had successfully demonstrated the therapeutic efficacies of these nanocarriers at the tumor site. MF-FA NPs thus had significant potential as promising nano-platforms for targeting delivery of dual chemotherapy agents and effective tumor treatment method.

## 4. Conclusions

In summary, we successfully fabricated a multi-functional FA targeting nanocarrier delivery system for co-delivery of hydrophilic chemotherapeutic drug GEM and hydrophobic PTX for the breast cancer treatment. The intelligent nanoparticles demonstrated outstanding stability and better biocompatibility. Additionally, the nanoparticles presented favorable tumor targeting capability *via* a folate receptor-mediated endocytosis and rapidly released drug in the acidic tumor microenvironment, thereby increasing the efficacy of anticancer drugs *in vivo*. Therefore, it was expected that the multi-functional MF-FA NPs would establish a promising platform for combination therapy to improve therapeutic efficacy.

## Conflicts of interest

The authors declare that they have no competing interests.

## Supplementary Material

RA-009-C9RA00276F-s001
