# Supplementary material for: Co-delivery of paclitaxel and gemcitabine via a self-assembling nanoparticle for targeted treatment of breast cancer
Source: RSC Adv. 2019 Feb 13;9(10):5512–20. doi: 10.1039/c9ra00276f (PMC9060788; doi:10.1039/c9ra00276f)
Supplement: RA-009-C9RA00276F-s001 [file RA-009-C9RA00276F-s001.pdf]

Electronic Supplementary Material (ESI) for RSC Advances.
This journal is © The Royal Society of Chemistry 2019

Supporting Information

Co-delivery of paclitaxel and gemcitabine via a self-assembly nanoparticle for targeting treatment of breast cancer

Meng Lei[a], Sijia Sha[a], Xueyuan Wang[b], Jia Wang[d], Xiao Du[c,*], Hang Miao[a], Hui Zhou[b], Enhe Bai[b], Jingmiao Shi[d], Yongqiang Zhu[b,*]

[a] College of Science, Nanjing Forestry University, No. 159 Longpan Road, Nanjing 210037, PR China

[b] College of Life Science, Nanjing Normal University, No. 1 Wenyuan Road, Nanjing 210037, PR China

[c] Department of Pharmaceutics, School of Pharmacy, China Pharmaceutical University, Nanjing 210009, PR China

[d] Jiangsu Chia Tai Fenghai Pharmaceutical Co. Ltd., No. 9 Weidi Road, Nanjing 210046, PR China

E-mail: zhyqscu@hotmail.com; duxiaojianai@126.com

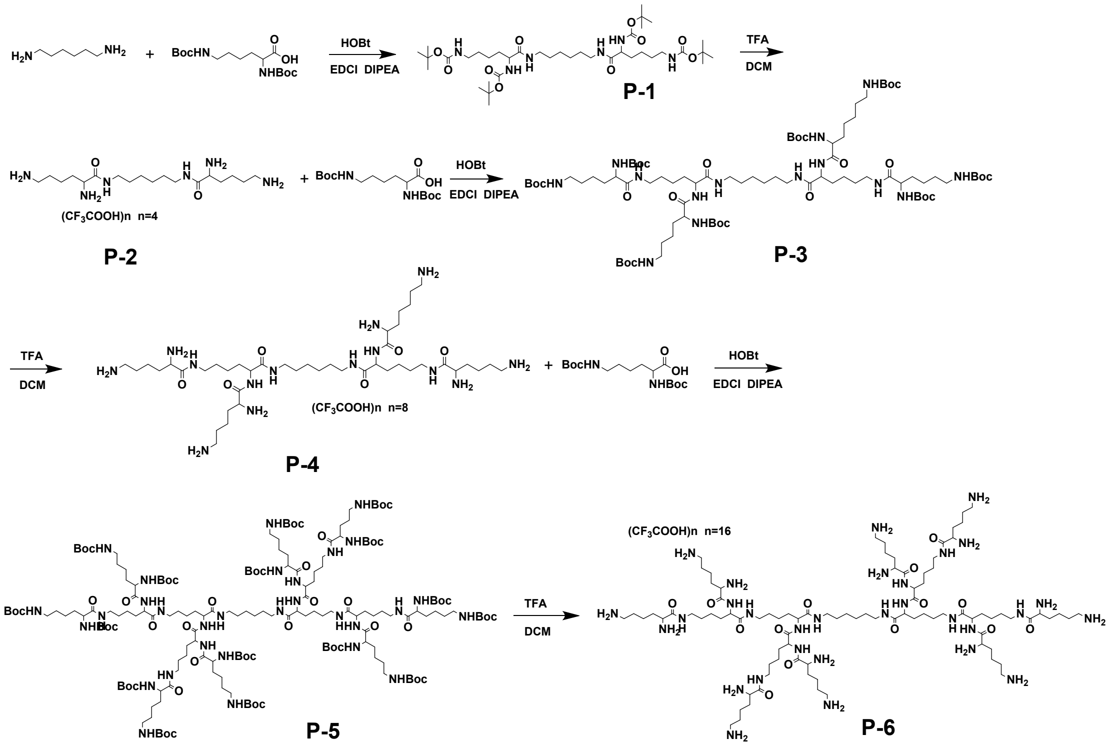

Fig. S1 Synthesis of P-6.

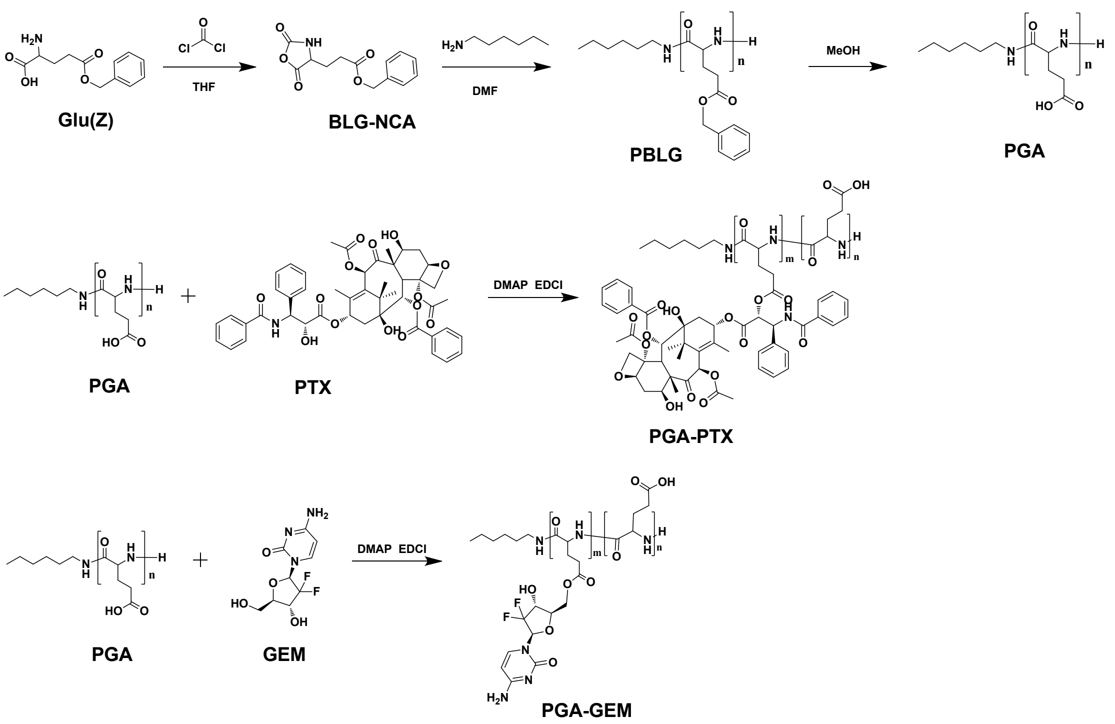

Fig. S2 Synthesis of PGA, PGA-PTX and PGA-GEM.

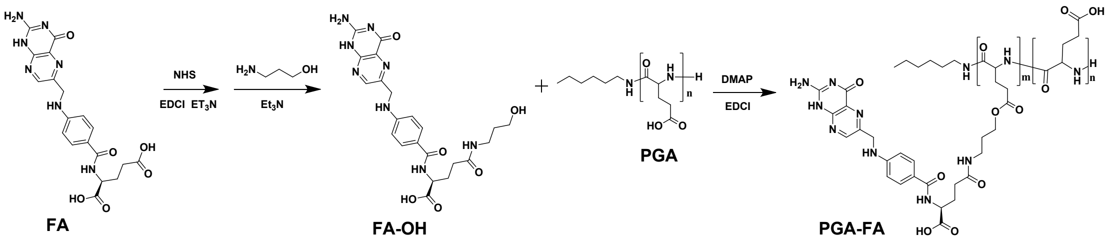

Fig. S3 Synthesis of PGA-FA.

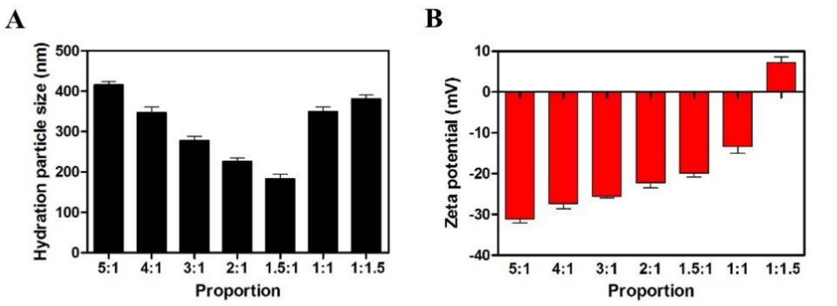

Fig. S4 Hydration particle size (A), and zeta potential (B) of different proportions in MF-FA NPs.
